# Dear Computer on My Desk, Which Candidate Fits Best? An Assessment of Candidates’ Perception of Assessment Quality When Using AI in Personnel Selection

**DOI:** 10.3389/fpsyg.2021.739711

**Published:** 2021-10-21

**Authors:** Jessica Schick, Sebastian Fischer

**Affiliations:** Business Psychology, Hamm-Lippstadt University of Applied Sciences, Hamm, Germany

**Keywords:** artificial intelligence, vignette study, personnel selection, candidate perception, intelligent recruitment, HR

## Abstract

Recently, with the increase in technological capabilities and the need to reduce bias in candidate selection processes, artificial intelligence (AI)-based selection procedures have been on the rise. However, the literature indicates that candidate reactions to a selection process need to be considered by organizations that compete for employees. In this study, we investigate reactions to AI-based selection procedures in a three-dimensional vignette study among young adults in Germany. By investigating the effects of the dimensions of AI complexity, intangibility, and reliability on the perceived quality of assessment of potential candidates, we found that AI complexity and intangibility impact the perceived quality of assessment negatively when the candidates’ knowledge, strengths, and weaknesses should be assessed. We also found interactive relationships of all three dimensions for the assessment of motivation. In sum, results indicate that candidates are skeptical toward the assessment quality of AI-intense selection processes, especially if these assess complex assessment criteria such as personality or a job performance forecast. Hence, organizations need to be careful when implementing AI-based selection procedures. HR implications are made on the basis of these results to cope with negative candidate perceptions.

## Introduction

Five years ago, no one would have imagined being interviewed and evaluated by AI while applying for a job. What seemed fairly strange in the past is now becoming more realistic (Ryan and Ployhart, 2014). AI can be defined as “a system’s ability to interpret external data correctly, to learn from such data, and to use those learnings to achieve specific goals and tasks through flexible adaptation” (Kaplan and Haenlein, 2019, p. 15). As such, AI is suggested to improve applicant selection procedures, as it may reduce human judgment bias.

With companies discovering the utility of AI in applicant selection, a question arises of whether the applicants perceive value in being assessed by AI as well (Woods et al., 2020). The process of candidate selection marks the beginning of the company–employee relationship, and perceptions of future employees concerning the quality of assessment in an AI-based assessment process may matter for companies that wish to attract employees (e.g., Langer et al., 2021a). In fact, the applicants’ perceptions of the selection process influence the outcomes, such as the impression of the organization’s justness (Schinkel et al., 2013); job performance (McCarthy et al., 2013); and attitudes, intentions, and behaviors (McCarthy et al., 2017). During the selection process, the candidates’ perceptions of the assessment are particularly important (Hausknecht et al., 2004). Candidates’ perception of assessment is defined as the way the candidates think and feel about the hiring process, of being tested and judged (Ryan and Ployhart, 2000). With detailed information on the assessment process being usually inaccessible for candidates, it is specifically the face validity and perceived predictive validity of the process that are considered strong predictors of the candidate’s perceptions, including their attitude toward the tests and selection process (Hausknecht et al., 2004). Drawing from this work, we conceptualize assessment quality perceptions as an extension to which the assessment process is perceived as detailed and thorough.

The aim of this study was to provide insights into assessment quality perceptions in various AI assessment situations, all in a personnel selection scenario. While using AI in personnel selection has been on the rise and has become increasingly popular among recruiters in the field of HR (Upadhyay and Khandelwal, 2018; Woods et al., 2020), empirical evidence points to the fact that applicants may not view AI in personnel selection favorably (e.g., Blacksmith et al., 2016; Wesche and Sonderegger, 2021). For example, applicants may feel that if personal exchange reduces, they have fewer opportunities to perform (Langer et al., 2020). Moreover, such a personal exchange may provide a less fruitful information to the applicant, and perceptions of job-relatedness lower compared with face-to-face interviews (Sears et al., 2013). Technological interviews brought about less favorable evaluations of the interviewer (Sears et al., 2013), and applicants feel greater uncertainty during the interview (Mirowska and Mesnet, 2021). In this research, we want to investigate the role of AI for such negative reactions in more detail. Specifically, we want to focus on different dimensions of AI, AI complexity, AI intangibility, and AI reliability, drawn from the literature on technology adoption (e.g., Lankton et al., 2014). We suggest that different dimensions of AI influence applicant reactions to the selection process. We test our predictions in a 3 (AI complexity)×3 (AI intangibility)×3 (AI reliability) factorial vignette study. Thereby, we add to the nascent literature on applicant reactions to AI based selection procedures by providing a generalizable framework to conceptualize AI in personnel selection and evidence on the relative importance of the dimensions of AI when assessing different assessment criteria.

## Theoretical Background

### Candidates’ Views on the Assessment Quality

Mentioned previously, the literature suggests that AI in an assessment process reduces applicants’ assessment quality perceptions (e.g., Blacksmith et al., 2016; Wesche and Sonderegger, 2021), in spite of potential benefits such as reduced bias (Quillian et al., 2017), or the potential to tap previously unknown sources of information (e.g., personality; Tengai AB, 2019). Woods et al. (2020) provide an overview of potential concerns that limit assessment quality perceptions ranging from concerns on the validity of the capabilities of the AI, potential technology-related biases, the applicant’s insufficient digital familiarity, to privacy concerns.

For different kinds of AI, these concerns apply in various degrees. For example, a candidate may be confident that AI can reliably pull the information on academic achievements from a resume while she may be less confident that AI can reliably draw the same information from information available in social networks. Therefore, we want to assess assessment quality for different criteria of assessment, similar to the assessment quality of non-AI selection processes.

We base our investigation on three criteria of assessment quality stemming from the work of Gilliland (1993) and Ostroff and Zhan (2012): the quality of assessment of candidates’ knowledge, motivation, and strengths and weaknesses. These three criteria represent key facets of candidates’ self-awareness and their opportunity to perform during the selection process (i.e., to self-evaluate and contribute to the selection process; Gilliland, 1993; Weekley and Ployhart, 2006; Ostroff and Zhan, 2012).

### Dimensions to Assess AI in an Automated Personnel Selection Process

AI for personnel selection consists of a variety of methods, and these evolve rapidly (e.g., Oswald et al., 2020; Tippins et al., 2021). Therefore, for investigating applicant reactions to AI in personnel selection, we grouped the various methods on three dimensions, which we drew from the literature on the adoption of new technology in general. In this field, research has shown that technology needs to be perceived as useful to be adopted by individuals and that usefulness can be described in the dimensions of functionality, helpfulness, and reliability (e.g., Lankton et al., 2014).

Based on the research by Lankton et al. (2014), and applied to AI, we define AI complexity as the extent to which AI is believed to be functional as it assesses either a narrow set or a wide range of criteria. AI intangibility is the perception that AI is helpful as it assesses a difficult-to-assess criterion vs. one that is more straightforward to assess. AI reliability is the perception that AI works properly and in a flawless manner under any given circumstance.

### AI Complexity

**Figure 1:**
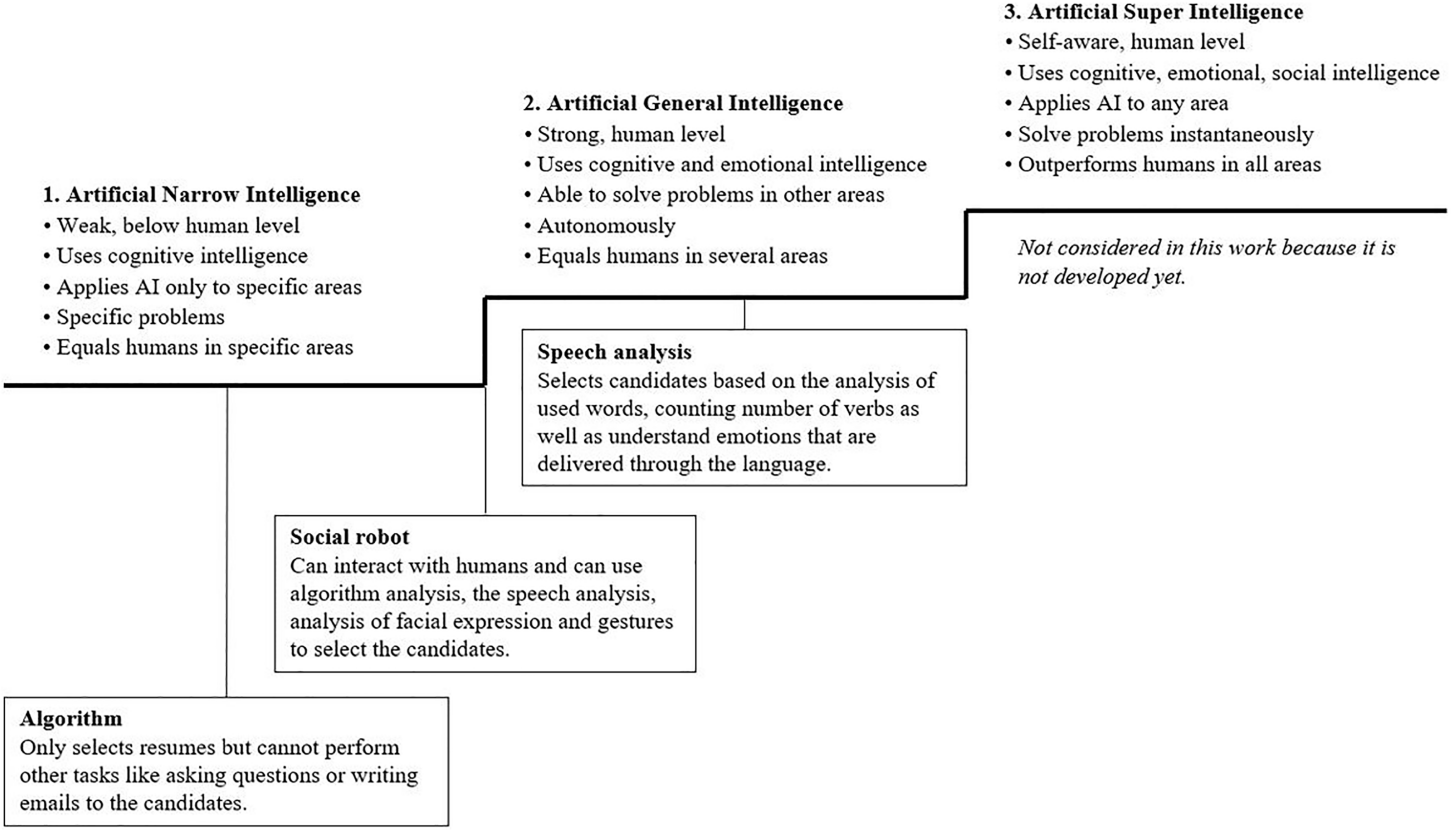
Classification of artificial intelligence and placement in HR. Classification of three AI stages is provided by Kaplan and Haenlein (2019).

AI can be classified depending on the degree of complexity (Meskó et al., 2018; Kaplan and Haenlein, 2019), with more intelligent systems potentially offering better functionality, as they can integrate more data. Kaplan and Haenlein (2019) developed an AI implication framework for organizations, which we have used to classify AI complexity in selection processes. The authors suggest that AI can be classified into artificial narrow intelligence, artificial general intelligence, and artificial superintelligence, as well as into different types of systems and competencies (Figure 1). In this study, we selected *algorithms*, *speech analysis*, and *robots for interviews* as we found existing applications of these methods on the market for personnel selection tools.

An *algorithm* is a procedure for solving tasks of a predefined specific class based on rules and experience, thus classified as narrow intelligence (Kaplan and Haenlein, 2019). In the field of HR, algorithms are used to preselect candidates through software like the “Applicant Tracking System” (Rietsema, 2019). For HR, this AI tool has the advantage of increasing the efficiency by saving time and resources during the repetitive tasks of reading resumes and checking the requirements.

*Speech analysis* analyzes communication beyond the algorithm by additionally measuring “hidden” information, such as the choice of words or the sentence structure that the candidate uses (Precire Technologies GmbH, 2020). The software uses algorithms but can additionally detect emotions and the personality. Precire (Precire Technologies GmbH, 2020), for example, predicts future customer relation skills and employee communication skills on the basis of linguistics.

*Social robots* are complex combinations and use different types of AI. Multiple types of these *robots* already exist to interview and select candidates while simulating human behavior, speech, and decision-making. An example for a social robot is Tengai (Tengai AB, 2019) who can speak, listen, confirm, and show emotions while assessing soft skills and personality traits during an interview. According to the developer, a valuable benefit of using *robots for interviews* is the elimination of human bias because the robots do not consider the appearance, age, gender or background of the candidates. Additionally, the selection process takes place in a data-driven manner and saves time.

Previous research has shown that AI-intensive selection is perceived to be of low quality because HR are unable to provide satisfactory explanations regarding the evaluations of AI systems due to the highly technical nature of AI (Booth et al., 2017). Aside from reducing fairness perceptions, this may also lower the perceptions of quality as the assessment occurs in a “black box” and may include only those aspects of the interaction between an applicant and the company that are readily evaluable. Thus, we predict that the more complex the AI is, the less comprehensible the result will be. Therefore, the *robot interview* assessment would be perceived to have a lower assessment quality than that of *speech analysis* and *algorithms*.

*Hypothesis*1: AI complexity has an impact on the candidates’ perception of assessment quality such that more complex forms influence the candidates’ perceptions negatively.

### AI Intangibility

The overall objective of the selection process was to find the best candidate for a position, the person who has the best chances to perform well. To reach that goal, recruiters investigate numerous criteria during the selection process, such as the candidates’ knowledge, skills, experience, and personality (Bisani, 1989). Generally, criteria vary in tangibility, with some being more difficult to assess than others. For example, knowledge, experience, and skills are easy to assess by investigating the candidate’s curriculum vitae, whereas the literature indicates that personality is relatively hard to assess. This is because there is no gold standard for assessing personality that may overcome obstacles like applicants faking (Morgeson et al., 2007).

In this study, we suggest that AI would be most helpful for HR if it can assess difficult criteria, such as *personality*, or even perform a more difficult task, such as providing a *job performance forecast*. However, from the candidates’ viewpoint, they may find the tool most trustworthy if it supports HR in compiling available information on *skills* rather than analyzing other potentially sparse or incomplete data for cues on personality of future job holders. Thus, we suggest that, with the increased complexity of AI, the candidates will perceive the recruitment process to be of a low quality. In conclusion, intangibility can have a negative effect on candidates’ perception of the assessment quality.

*Hypothesis*2: AI intangibility has an impact on candidates’ perception of assessment quality such that intangibility has a negative effect on the candidates’ perception.

### AI Reliability

When submitting applications, candidates share personal information and must trust that AI processes their data without errors. Intelligent systems are already capable of diagnosing X-ray images as competent as or even better than a physician’s diagnosis. Nevertheless, those concerned frequently report difficulties in trusting the judgement of a machine (Kaplan and Haenlein, 2019). Generally, users prefer to scrutinize technology or at least know that it is (or has been) carefully evaluated by a human, especially if the system makes far-reaching decisions (Wang et al., 2006). In terms of personnel selection, AI falls into this category.

For any technology, Giffin (1967) suggested that expertness is a dimension that leads to trust and that trust is based on other factors, such as reliability and activeness (Giffin, 1967). We manipulate reliability by indicating flawlessness through three degrees of usage: the AI system may be either *new*, *tried and true*, or *approved*. *Approved* reflects a higher degree of reliability with clues of security, expertness, and activeness than that of *new*, as well as a higher degree than that of *tried and true*.

*Hypothesis*3: AI reliability has an impact on candidates’ perception of assessment quality such that the reliability of a system has a positive effect on the candidates’ perception.

### Interactive Effects of System Functioning

First, we suggest that the assumed negative impacts of AI complexity and intangibility can be balanced by the assumed positive impact of high AI reliability. The negative effect of *robotic interview forecasting job performance* (a low-quality combination, according to our hypotheses) becomes weaker when the system is described as *approved*. Thus, the interaction of high AI complexity, high AI intangibility, and high AI reliability leads to a higher perception of assessment quality.

Second, it may be estimated that the assumed negative impacts of high AI complexity and low AI reliability in a system can be balanced by low AI intangibility. To be more precise, the negative effect of a *new robotic interview* becomes weaker when it is assessing *skills*, an evaluation objective that is supposedly rather straightforward. Therefore, the combination of high AI complexity, low AI reliability, and low AI intangibility in a system leads to a higher assessment quality perception.

Third, by looking at the presented estimated effects of AI complexity and reliability, it can be assumed that these effects are additive when aiming to achieve the highest perception of assessment quality. To be more concrete, an *approved algorithm* leads to a candidate’s highest quality perceptions. Thus, these assumptions lead to three plausible interactions stated in the following hypotheses.

*Hypothesis*4a: A combination of high AI complexity (e.g., robotic interview) and high AI intangibility (e.g., job performance forecast) will have a positive impact on candidates’ perception when the AI reliability in the system is high (e.g., approved).

*Hypothesis*4b: A combination of high AI complexity (e.g., robotic interview) and low AI intangibility (e.g., skill) will have a positive impact on candidates’ perception when the AI reliability in the system is low (e.g., new).

*Hypothesis*4c: The influences of AI complexity and reliability in a system are additive; that is, a combination of algorithm and an approved system will lead to the most positive candidate perception of assessment quality.

**Figure 2:**
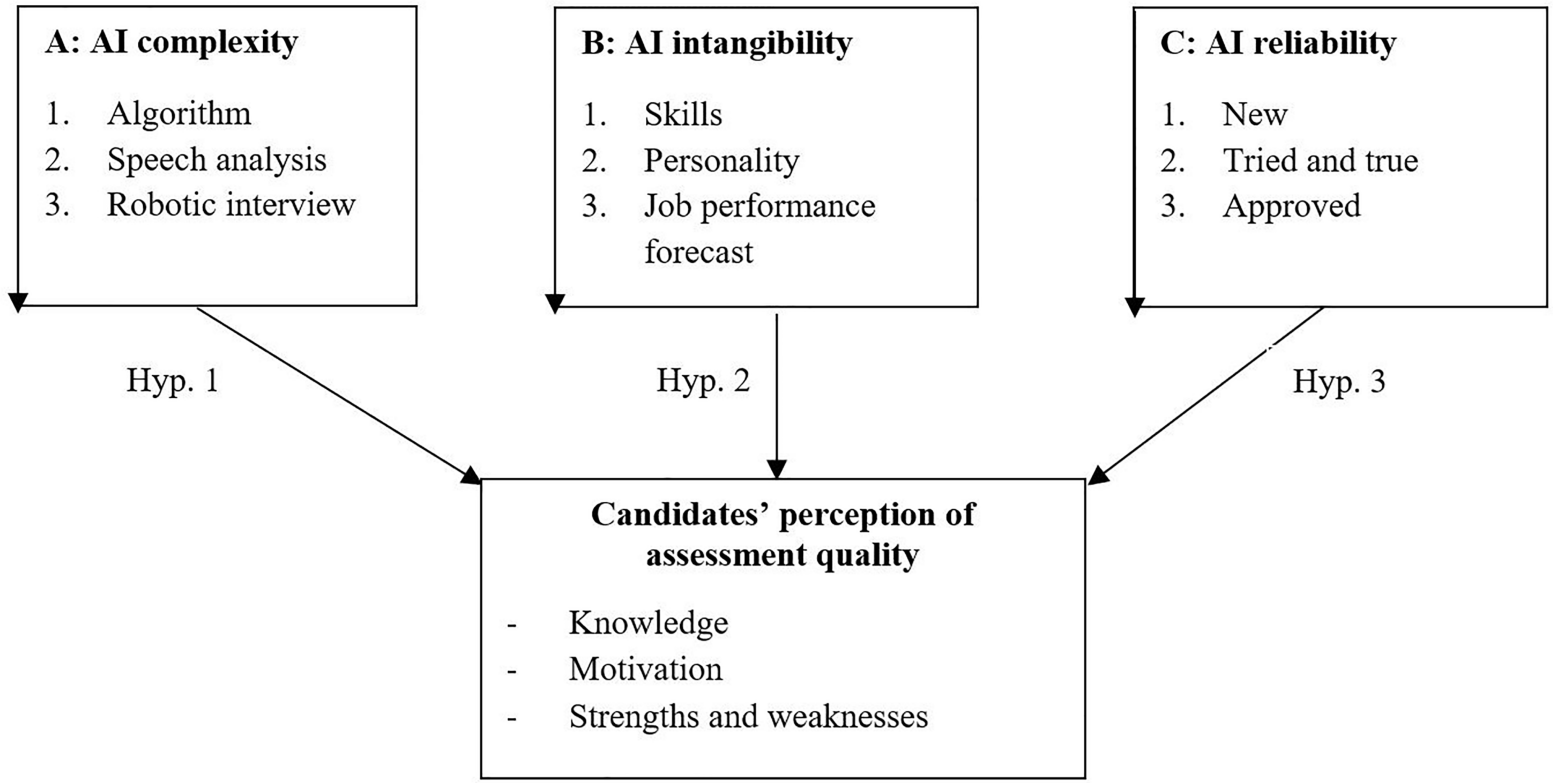
Model illustration.

For a comprehensive model overview of this work, an illustration is presented in Figure 2.

In addition to our tests of the hypotheses outlined above, we provide descriptive information on the candidates’ expectations on AI usage in future selection.

## Materials and Methods

### Participants

The target population for our study were current students aged 18 and 28. We chose this target group as it grew up in the time of digitalization and is likely to enter the job market within the next three to 5years; hence, they are potentially set to deal with such technology.

Individuals were invited to participate in an online survey using the Questback Unipark software through postings in chat groups, LinkedIn, direct messages, and clues in lectures. In sum, roughly 200 individuals were approached with the survey. However, the exact response rate is difficult to assess, as some contacted individuals may not have been eligible to participate in the survey. Out of a total of *N*=129 participants who started the survey, 22 opted out during the survey and 11 violated the quotes, yielding a final sample size of *N*=96 participants (53% females, 44% males, and 3% diverse).^1^ A large proportion of participants were in their early twenties and studied business or health-related subjects (e.g., business administration, psychology, medicine, sport). The study language was German. The data were collected between January and March 2020. On average, it took around 6min to complete the survey.

### Procedure

We assessed candidates’ assessment quality perceptions in a cross-sectional vignette study. The vignettes consist of the three dimensions for technology usefulness as independent factors (AI complexity, AI intangibility, AI reliability), whereby each factor has three characteristics (levels) that are combined in the scenario, leading to a 3×3×3 vignette design. All the possible levels of each factor were combined with each other so that the total vignette population equals 27. To reduce repetitiveness, and in line with the recommendations of the literature (e.g., Auspurg et al., 2009), only a randomly selected subset of five vignettes was shown to each participant.

**Figure 3:**
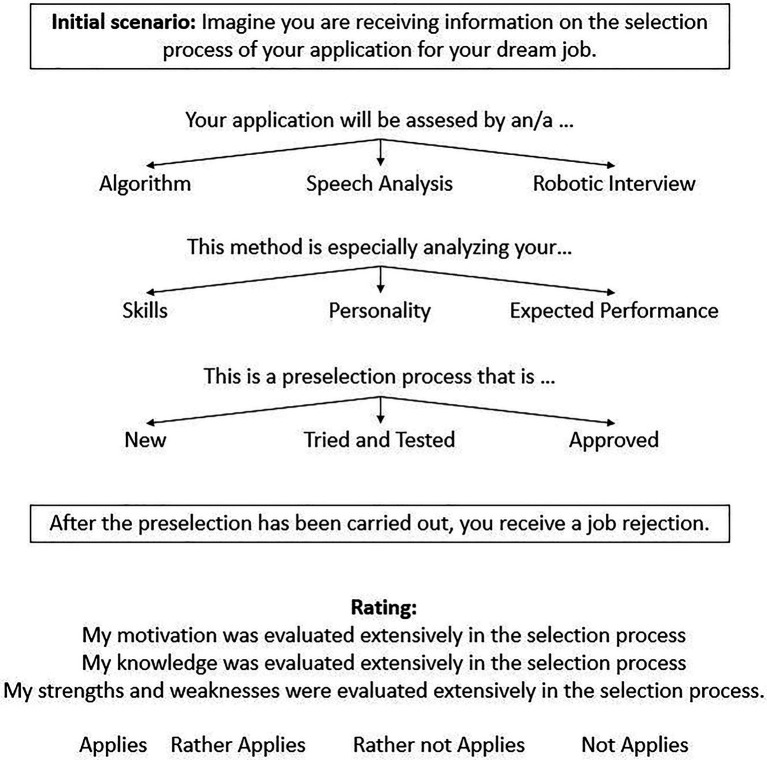
Vignette examples.

Before presenting the vignettes, we presented an initial situation description to all participants, asking them to imagine the application for their dream job. After sending the application, they were informed that the first step of the selection process is done using AI. Following this situation, a random set of five of the 27 vignettes were presented to each participant (i.e., randomized formation of vignette sets). The vignettes asked the participants to put themselves in the situation of receiving information about the selection process and its outcome. No definitions of the levels of AI used in the process were provided. Each vignette ended with a rejection decision. An illustration of the vignette design is shown in Figure 3.

After each vignette, the participants were asked to assess the perceived *assessment quality* with three items. These items evaluate the perception regarding the assessed *knowledge*, *motivation*, and *strengths and weaknesses*. A sample item was “*My knowledge was extensively evaluated in the preselection process*.” The three items are rated with a verbalized 4-point rating scale with the following categories: *true* (1), *rather true* (2), *rather not true* (3), and *not true* (4).

After each participant completed all five vignettes, we asked them to rank the three presented AI complexities according to their own preferred use. Each participant estimated the use of AI in future personnel selection processes. Then, they assessed their perceived level of preparedness for the use of AI in a selection process. Finally, they were asked whether they have already been exposed consciously to AI in the selection process.

We analyzed the data using multivariate analysis of variance (MANOVA) in SPSS 25.0.0.

## Results

Along with a series of *post hoc* analyses using least significant difference, MANOVA was performed to examine the individual mean difference comparisons across all three levels of the three factors and three dependent variables of assessment quality. Statistically significant MANOVA effects were obtained for AI complexity [Pillai’s trace=0.09, *F*(6, 904)=7.11, *p*<0.01; Table 1], with an effect size of *ηp*^2^=0.045, implying that approximately 4.5% of the variance of the dependent variable is associated with the factor of AI complexity. Moreover, for AI intangibility [Pillai’s trace=0.04, *F*(6, 904)=3.13, *p*<0.01], a significant main effect of *ηp*^2^=0.02 was observed. The three-way interaction of AI complexity, intangibility, and reliability was found to reach marginal significance [Pillai’s trace=0.074, *F*(24, 1,359)=1.43, *p*<0.10, with an effect size of *ηp*^2^=0.025], implying that approximately 2.5% of the variance of the dependent variable is associated with the interaction of the three factors.

**Table 1:** Multivariate tests.

Predictor	Pillai’s trace	*F*	Hyp. df	Error df	Sig.	*ηp* ^2^
(Constant)	0.955	3173.436	3.000	451.000	0.000	0.955
AI complexity	0.090^**^	7.113	6.000	904.000	0.000	0.045
AI intangibility	0.041^**^	3.133	6.000	904.000	0.005	0.020
AI reliability	0.009	0.650	6.000	904.000	0.690	0.004
AI intelligence ^*^ AI intangibility	0.017	0.654	12.000	1359.000	0.797	0.006
AI complexity ^*^ AI reliability	0.015	0.586	12.000	1359.000	0.855	0.005
AI intangibility ^*^ AI reliability	0.021	0.797	12.000	1359.000	0.654	0.007
AI complexity ^*^ AI intangibility ^*^ AI reliability	0.074	1.429	24.000	1359.000	0.082	0.025

Note.

* *Sig. <p=0.05*;

** *Sig. <p=0.01*.

To detect the differences, results of intercorrelation were examined (shown in Table 2). For a better overview, the results of the hypothesis testing are summarized in Table 3.^2^

**Table 2:** Tests of inter-subject effects.

Source	Dependent variable	df	*F*	Sig.	*ηp* ^2^
Model	Motivation	26	1.418	0.085	0.075
	Knowledge	26	2.378	0.000	0.120
	Strengths and weaknesses	26	1.523	0.049	0.080
(Constant)	Motivation	1	7249.613	0.000	0.941
	Knowledge	1	4174.470	0.000	0.902
	Strengths and weaknesses	1	5787.203	0.000	0.927
AI complexity	Motivation	2	1.434	0.239	0.006
	Knowledge	2	10.429^**^	0.000	0.044
	Strengths and weaknesses	2	6.106^**^	0.002	0.026
AI intangibility	Motivation	2	0.235	0.790	0.001
	Knowledge	2	5.750^**^	0.003	0.025
	Strengths and weaknesses	2	3.903^*^	0.021	0.017
AI reliability	Motivation	2	0.716	0.489	0.003
	Knowledge	2	0.421	0.657	0.002
	Strengths and weaknesses	2	1.666	0.190	0.007
AI complexity * AI intangibility	Motivation	4	0.195	0.941	0.002
	Knowledge	4	1.757	0.136	0.015
	Strengths and weaknesses	4	0.264	0.901	0.002
AI complexity ^*^ AI reliability	Motivation	4	0.401	0.808	0.004
	Knowledge	4	1.029	0.392	0.009
	Strengths and weaknesses	4	0.253	0.908	0.002
AI intangibility ^*^ AI reliability	Motivation	4	1.356	0.248	0.012
	Knowledge	4	0.471	0.757	0.004
	Strengths and weaknesses	4	1.063	0.374	0.009
AI complexity ^*^ AI intangibility ^*^ AI reliability	Motivation	8	2.886^**^	0.004	0.048
	Knowledge	8	1.383	0.202	0.024
	Strengths and weaknesses	8	1.217	0.287	0.021

Note.

* *Sig. <p=0.05*;

** *Sig. <p=0.01*.

**Table 3:** Overview of hypothesis testing.

Hypothesis 1	AI complexity has an impact on candidates’ perception of extensive assessments such that complexity has a negative effect on the candidates’ perception.	Partly confirmed 2/3	Knowledge, strengths and weaknesses
Hypothesis 2	AI intangibility has an impact on candidates’ perception of extensive assessments such that intangibility has a negative effect on the candidates’ perception.	Partly confirmed 2/3	Knowledge, strengths and weaknesses
Hypothesis 3	AI reliability has an impact on candidates’ perception of extensive assessments such that reliability has a positive effect on the candidates’ perception.	Rejected 0/3	—
Hypothesis 4b	A combination of high complex AI (e.g., robotic interview) and low AI intangibility (e.g., skills) will have a positive impact on candidates’ perception when the reliability in the system is low (e.g., new).	Partly confirmed 1/3	Motivation
Hypothesis 4c	The influences of AI complexity and reliability in a system are additive. In other words, a combination of algorithm and an approved system will lead to the most positive candidate perception of extensive assessments.	Partly confirmed 1/3	Motivation

3/3=independent variables: knowledge, motivation, and strengths and weaknesses.

Hypothesis 1 predicts that AI complexity affects the candidates’ perception of the assessment quality such that complexity has a negative effect on the candidates’ perception. The results indicate that AI complexity is statistically significant related to the perception of *knowledge* [*F*(2, 453)=10.43, *p*<0.01, *ηp*^2^=0.044] and *strengths and weaknesses* [*F*(2, 453)=6.11, *p*<0.01, *ηp*^2^=0.026]. However, it was found that AI complexity exhibits no significant relationship with the perception of *motivational* assessment [*F*(2, 453)=1.43, *p*>0.05, *ηp* ^2^=0.006].

The results of the *post hoc* test indicate a significant difference among the levels of complexity when assessing *knowledge* and *strengths and weaknesses*. Taken together, when assessing *knowledge*, the *algorithm* was perceived as a more extensive assessment method than the *robotic interview* (*MD*=−0.21, *p*<0.05) and *speech analysis* (*MD*=−0.43, *p*<0.01). When *strengths and weaknesses* were assessed, the levels of *algorithm* and *speech analysis* were not found to be significantly different from each other. *Robotic interview* was perceived on average as a lower-quality assessment method than *algorithm* (*MD*=0.27, *p*<0.05) and *speech analysis* (*MD*=0.25, *p*<0.05). This means that the complex AI method *robotic interview* indeed has a negative impact on the candidates’ perception when evaluating *strengths and weaknesses*. Thus, Hypothesis 1 can be partly confirmed.

Hypothesis 2 predicts that AI intangibility affects the candidates’ perception of the assessment quality such that intangibility has a negative effect on the candidates’ perception. The results indicate a significant relationship between AI intangibility and the perception of *knowledge* [*F*(2, 453)=5.75, *p*<0.01, *ηp*^2^=0.025] and *strengths and weaknesses* [*F*(2, 453)=3.90, *p*<0.05, *ηp*^2^=0.017]. However, it was found that AI intangibility does not exhibit a significant relationship with the perception of *motivational* assessment [*F*(2, 453)=0.24, *p*>0.05, *ηp*^2^=0.001].

*Post hoc* comparisons for AI intangibility revealed no significant difference between the levels of *skills* and *job performance forecast* when assessing *knowledge* and *strengths and weaknesses*. In both cases, the evaluation of *personality* was perceived as a lower assessment quality than that of *skills* (knowledge: *MD*=0.22, *p*<0.01; strengths and weaknesses: *MD*=0.23, *p*<0.01) and *job performance forecast* (knowledge: *MD*=0.35, *p*<0.05; strengths and weaknesses: *MD*=0.21, *p*<0.05). Taken together, when *knowledge* and *strengths and weaknesses* were assessed, the participants perceived that the evaluation of *personality* is the lowest assessment quality given the three presented options. Therefore, Hypothesis 2 is rejected.

Hypothesis 3 predicts that AI reliability affects the candidates’ perception of the assessment quality such that the degree of reliability of a system has a positive effect on the candidates’ perception. The results indicate that AI reliability does not exhibit a significant relationship with the different perceptions measured by the assessment of *motivation* [*F*(2, 453)=0.72, *p*>0.05, *ηp*^2^=0.003], *knowledge* [*F*(2, 453)=0.42, *p*>0.05, *ηp*^2^=0.002], and *strengths and weaknesses* [*F*(2, 453)=1.67, *p*>0.05, *ηp*^2^=0.007]. Looking back on the *post hoc* test, no significant difference between the factor levels can be demonstrated, concluding that Hypothesis 3 is rejected.

Hypothesis 4a states that a combination of high AI complexity (e.g., *robotic interview*) and intangible AI (e.g., *job performance forecast*) will have a positive impact on candidates’ perception when the AI reliability in a system is high (e.g., *approved*). Slope difference test indicates a significant slope difference between a *performance forecast* with an *approved* system and a *performance forecast* with a *new* system (slope difference=−0.28, *t*-value=−3.20, *p*≤0.01). This means that a high degree of AI reliability leads to a less negative impact of high AI complexity and high AI intangibility. The outcome is a higher perception of assessment quality when assessing candidates’ motivation. Therefore, Hypothesis 4a can be partly supported.

Hypothesis 4b predicts that a combination of high AI complexity (e.g., *robotic interview*) and low AI intangibility (e.g., *skill*) will have a positive impact on candidates’ perception when the AI reliability in a system is low (e.g., *new*). Slope difference test indicates a significant slope difference between a *performance forecast* with a *new* system and the evaluation of *skills* with a *new* system (slope difference=0.28, *t*-value=3.22, *p*≤0.01). This indicates that low AI intangibility leads to a less negative impact of high AI complexity and low AI reliability. This interaction leads to a higher perception of assessment quality when assessing motivation. Therefore, Hypothesis 4b is partly supported.

Hypothesis 4c states that the influences of AI complexity and reliability are additive. In other words, a combination of *algorithm* and an *approved* system will lead to the most positive candidate perception of the assessment quality. Slope difference test indicates a significant difference between a *performance forecast* with an *approved* system and the evaluation of *skills* with an *approved* system (slope difference=−0.20, *t*-value=−2.37, *p*≤0.05). This indicates that the combination of low AI complexity and high AI reliability leads to a higher perception of *motivation* assessment independent of AI supportiveness. Thus, Hypothesis 4c is partly supported.

### Further Findings

The means of the levels among factors were examined. In total, the best vignette that was rated among the vignette population assessed *knowledge* using a *tried and true algorithm* to *forecast job performance* (*M*=2.00, *SD*=0.59). The best vignette assessing *knowledge* states that “the preselection is done by an algorithm that especially makes analyses for forecasting your job performance. This is a procedure for preselecting applicants that is currently being tried and true for this use. After the procedure has been carried out, you receive a rejection for your application.”

The best rated vignette for assessing *strengths and weaknesses* described the use of an *approved algorithm* that analyzes *skills* (*M*=2.47, *SD*=0.80). To assess *motivation*, the best described scenario used an *approved speech analysis* that *forecasts job performance* (*M*=2.53, *SD*=0.74).

The vignette in which the participants had the worst perception of assessment in total described the assessment of *motivation* using a *new algorithm* to analyze *skills* (*M*=3.38, *SD*=0.74). The worst vignette assessing *motivation* states that “the preselection is done by an algorithm that especially analyses your skills. This is a new procedure for preselecting applicants. After the procedure has been carried out, you receive a rejection for your application.” In the assessment of *strengths and weaknesses*, the worst vignette combined a *tried and true robotic interview* to analyze *personality* (*M*=3.36, *SD*=0.67). Finally, the worst combination for assessing *knowledge* used an *approved speech analysis* to analyze *personality* (*M*=3.08, *SD*=0.95).

### Additional Questions

This section focuses on the results of further questions. Participants were asked to rank AI methods according to which one they are likely to use themselves. To calculate the answer, Microsoft Excel (Microsoft Corp., Redmond, WA, United States) was used. The ranking results of the preferred AI complexity usage for assessment in selection are as follows: (1) *algorithm*, (2) *robotic interview*, and (3) *speech analysis*. Given these three methods, according to the results, the candidates would prefer to be assessed by an *algorithm* and least likely to prefer being assessed by *speech analysis* during a personnel selection process. This finding supports the results of MANOVA when *knowledge* is assessed. This is because, in MANOVA, *speech analysis* was the most unpopular method. When *strengths and weaknesses* were assessed, *speech analysis* was perceived as a better assessment method than *robotic interview*. In summary, offering no tradeoffs through direct ranking, *speech analysis* is the least preferred method.

The respondents estimated the use of AI in future application processes. The results show that 6.3% thought that AI will *always* be used, more than half (53.1%) thought that AI will *often* be used, around one third (30.2%) thought that AI will be *occasionally* used, and 10.4% thought that AI will be *rarely* used. None of the participants thought that AI will *never* be used in future selection processes. In other words, more than half of the participants predicted that AI will often be used by HR, and none of them predicted an HR future without AI.

Next, the participants provided insights into their preparation levels of AI use in the application process. The results indicate that 1.0% felt very well prepared, 12.5% felt well prepared, around one third (32.3%) felt neither well nor poorly prepared, 38.5% felt poorly prepared, and 15.6% felt very poorly prepared. This means that more than half of the respondents felt poorly or very poorly prepared for the future use of AI in personnel selection.

Finally, the participants provided insights into whether they have already dealt with AI in any application process. Less than 10% (9.4%) stated that they have come into contact with AI during a selection process. This result reveals the relatively low frequency of AI use in this study’s sample.

## Discussion

The aim of this study was to answer the question regarding the extent to which AI complexity, intangibility, and reliability impact candidates’ perception of assessment quality in selection processes. Our results generally align well with previous research in the field, as highest assessment quality is associated with reduced AI complexity and intangibility, and high reliability. Candidates seem to be skeptical towards complex AI, for which they may lack understanding (see for example Mirowska and Mesnet, 2021). Of note, system reliability is only relevant in association with the other dimensions of AI, indicating that the mere mentioning, that AI is reliable does not improve assessment quality perceptions. Potentially, this may be due to a lack of information on the functioning of the different AI methods in this study (see Langer et al., 2021b).

Investigating direct influences of the three dimensions in more detail, MANOVA revealed a significant negative impact of AI complexity on the assessment perception of knowledge and strengths and weaknesses (Hypothesis 1). A significant negative impact was also found for AI intangibility on the assessment perception of knowledge and strengths and weaknesses (Hypothesis 2). AI reliability did not influence candidate perceptions (Hypothesis 3). A comparison of effect sizes reveals the strongest effects for AI complexity.

Investigating, interactive influences of the three dimensions on assessment quality perceptions, MANOVA indicated that all three dimensions together influenced the perception of assessment quality of motivation. This revealed that a high degree of reliability combined with a high AI complexity and high AI intangibility leads to a higher perception of motivational assessment (Hypothesis 4a). Additionally, lower AI intangibility combined with a high AI complexity and low AI reliability results in a higher perception of motivational assessment (Hypothesis 4b). Finally, a combination of low AI complexity and high AI reliability leads to a higher perception of motivation assessment independent of AI intangibility (Hypothesis 4c). The perception of assessment quality was highest when the AI complexity used was algorithm, whereas it was lowest when speech analysis was used. This was supported through simple ranking of AI methods. The highest perception of AI intangibility was reached when the AI system forecasted job performance, and it was lowest when personality was evaluated. As predicted in the first three hypotheses, the best-rated vignette has a low AI complexity (algorithm), the lowest AI intangibility (skill), and the highest AI reliability (approved). Hence, we suggest that while AI may indeed be technically able to show HR managers the candidate that best fits the job, there is still some work to do to improve the candidates’ perception of such assessment practices. In addition to the above results, the results also indicate that there is no optimal combination of the studied dimensions in terms of candidate reactions.

A few findings stand out, as they do not support our hypotheses. The first unexpected finding was the nonsignificant impact of reliability in a system on candidates’ perception. Research has shown that the autonomous growing and diverse nature of AI has a critical impact on users’ trust, especially when handling very private data. The nonsignificant results indicate that the levels of AI reliability – *new, tried and true*, and *approved* – may be too subliminal and ambiguous because *new* systems are not necessarily worse than *approved* ones. Considering the studies of Wang et al. (2006) and Sears et al. (2013) and considering the doubts or skepticism toward new approaches, the negative impact of low reliability is still likely. Further research should focus on increasing the understanding of the factor reliability by researching technology’s characteristics that are attributed to that.

The second finding is that, with regard to AI intangibility, in this study, *personality* is perceived as the worst evaluation objective and not *job performance forecast*. While personality is a frequent evaluation objective in candidate selection, it often raises doubts among candidates (Rynes and Connerley, 1993; Rosse et al., 1994). Personality assessments may be troubling to candidates because business-related validity may be perceived to be low (Rynes and Connerley, 1993), while other evaluation objectives, such as *skills* and *job performance forecast*, may be more easily related to a job at hand. In conclusion, evaluating *personality* as an objective is perceived as a least extensive assessment among candidates.

Finally, we unexpectedly found interactive effects of the three dimensions only for *motivation* as a dependent variable, but not for *strengths and weaknesses* and *knowledge*. Additionally, the results indicate that the assessment of *motivation* is not significantly predicted by the three factors separately, but rather only by the interaction. *Motivation* is rather a fuzzy concept and may be categorized as more subjective to evaluate than *knowledge* or *strengths and weaknesses*. A potential reason is, that its indicators cannot be directly found on the resume in the form of study courses, skills, or missing experiences.

### Strengths, Limitations, and Future Research

Choosing a vignette design allowed us to manipulate more than one independent variable and detect interaction effects between the dimensions of technology usefulness. Thus, this research comes closer to reality because humans experience several influences simultaneously and usually face several tradeoffs when making judgements (Rost, 2018). Additionally, the scope of interpretation decreased as each participant received the same standardized initial situation as well as standardized scenario frame to base their judgement on (Alexander and Becker, 1978; Rost, 2018).

Nonetheless, a number of limitations exist. One limitation is the unnormal distribution of study backgrounds (technical vs. nontechnical). Each study background provides a different state of knowledge, touchpoints with AI, and different reasoning in perception. We accounted for this by including studies as a variable in our analyses. However, this did not influence the effect sizes we found for the dimensions of technology usefulness. Potentially, a larger sample size of technicians may have influenced our findings. For example, students in social studies may have a more negative perception because of the lack of personal human contact during the assessment, as well as less knowledge in systems functioning and skills. Future research should investigate the perception of students in specific study fields to reveal further effects.

A second limitation is the lack of reality in the vignettes. It may be difficult for the participants to imagine the vignettes without really experiencing the assessment process. To steer against these weaknesses, the dimensions were presented in bold, and the scenarios were kept short, realistic, and simple. Nevertheless, reality can be improved in future studies, for example, by providing pictures or video vignettes of the assessment situation. Such materials may facilitate the rethinking and imagination processes.

For further research, it would be interesting to find out whether the perception of extensive selection would change if the participants were accepted. In this study, all the participants received a rejection decision to standardize the conditions. Additional topics for future research may include a more in-depth investigation of the role of specific words (in this research, the *algorithm* was perceived particularly well by the participants), or a view of minority applicants who may react positively to bias-free assessment methods.

The results of this study suggest that if HR managers are planning to use complex AI methods, such as robots, and they want to evaluate intangible objectives, they should provide cues of high system reliability. As described above, this can be done, for example, by including expert opinions and providing test trials and enough details to understand the process. However, if HR managers wish to use complex AI methods but cannot guarantee a high degree of reliability, we recommend sticking to a less intangible evaluation objective, such as skills. Only if highly complex AI is associated with high reliability would HR managers be free to choose the evaluation objectives without weakening the assessment perception.

These results should not discourage the use of AI in assessment processes. An impressive finding is that none of the respondents could imagine a future without AI in personnel selection, with even strong predictions of heavy use. When this is connected to the presented findings, HR must improve the candidates’ perceived extensiveness before getting enchanted by the benefits of AI. An increase in technical development does not necessarily mean an increase in candidates’ perception. Students in 2021 may need more touchpoints and greater knowledge to feel comfortable and trust machines with decisions regarding their future personal lives.

## Data Availability Statement

The raw data supporting the conclusions of this article will be made available by the authors, without undue reservation.

## Ethics Statement

Ethical review and approval was not required for the study on human participants in accordance with the local legislation and institutional requirements. The patients/participants provided their written informed consent to participate in this study.

## Author Contributions

JS developed the research question and survey, and performed the statistical analyses. JS and SF conducted the literature search and drafted the article. All authors contributed to the article and approved the submitted version.

## Conflict of Interest

The authors declare that the research was conducted in the absence of any commercial or financial relationships that could be construed as a potential conflict of interest.

## Publisher’s Note

All claims expressed in this article are solely those of the authors and do not necessarily represent those of their affiliated organizations, or those of the publisher, the editors and the reviewers. Any product that may be evaluated in this article, or claim that may be made by its manufacturer, is not guaranteed or endorsed by the publisher.

## Footnotes

^1^Note that the sample size in the provided result tables is *N* = 480 because each participant rated five vignettes (96â€‰Ã— 5).

^2^Results were also investigated including a number of dichotomous control variables. No meaningful changes in effect sizes were found for age (young vs. old), studies (technical vs. non-technical) and experience with AI in selection (yes vs. no).
